# Retinal Microvascular Biomarker Assessment With Automated Algorithm and Semiautomated Software in the Montrachet Dataset

**DOI:** 10.1167/tvst.14.3.13

**Published:** 2025-03-12

**Authors:** Pétra Eid, Abderrahmane Bourredjem, Atif Anwer, Catherine Creuzot-Garcher, Pearse Andrew Keane, Yukun Zhou, Siegfried Wagner, Fabrice Meriaudeau, Louis Arnould

**Affiliations:** 1 Ophthalmology Department, Dijon University Hospital, Dijon, France; 2 Centre des Sciences du Goût et de l'Alimentation, AgroSup Dijon, CNRS, INRAE, Université Bourgogne, Dijon, France; 3 CIC 1432, Epidémiologie Clinique, Centre Hospitalier Universitaire Dijon-Bourgogne, Dijon, France; 4 Institut de Chimie Moléculaire Université de Bourgogne (ICMUB), Imagerie Fonctionnelle et moléculaire et Traitement des Images Médicales (IFTIM), Burgundy University, Dijon, France; 5 NIHR Biomedical Research Centre at Moorfields Eye Hospital NHS Foundation Trust, London, UK; 6 Institute of Ophthalmology, University College London, London, UK; 7 Pathophysiology and Epidemiology of Cerebro-Cardiovascular Diseases (PEC2), (EA 7460), Faculty of Health Sciences, Université de Bourgogne, Dijon, France

**Keywords:** automorph, siva, retinal fundus photographs, retinal microvascular biomarkers, retinal imaging, fundus photographs

## Abstract

**Purpose:**

To compare automated and semiautomated methods for the measurement of retinal microvascular biomarkers: the automated retinal vascular morphology (AutoMorph) algorithm and the Singapore “I” Vessel Assessment (SIVA) software.

**Methods:**

Analysis of retinal fundus photographs centered on optic discs from the population-based Montrachet Study of adults aged 75 years and older. Comparison and agreement evaluation with intraclass correlation coefficients (ICCs) between SIVA and AutoMorph measures of the central retinal venular and arteriolar equivalent, arteriolar–venular ratio, and fractal dimension.

**Results:**

Overall, 1069 fundus photographs were included in this study. The mean age of the patients was 80.04 ± 3.94 years. After the image quality grading process with an optimal threshold, the lowest rejection rate was 51.17% for the AutoMorph analysis (*n* = 522). The measure of agreement between SIVA and AutoMorph retinal microvascular biomarkers showed a good correlation for vascular complexity (ICC, 0.77–0.47), a poor correlation for vascular calibers (ICC, 0.36–0.23), and no correlation for vascular tortuosity. Significant associations between retinal biomarkers and systemic variables (age, history of stroke, and systolic blood pressure) were consistent between SIVA and AutoMorph.

**Conclusions:**

In this dataset, AutoMorph presented a substantial rejection rate. SIVA and AutoMorph provided well-correlated measurements of vascular complexity and caliber with consistent clinical associations. Further comparisons are needed before a transition is made from semiautomated to automated algorithms for the analysis of retinal microvascular biomarkers.

**Translational Relevance:**

Open source software needs to be compared with former semiautomated software for retinal microvascular biomarkers assessment before transition in daily clinic and collaborative research.

## Introduction

Quantitative analysis of retinal microvascular biomarkers based on retinal fundus photographs has been extensively performed since the early 2000s. Semiautomated software applications such as Singapore “I” Vessel Assessment (SIVA; National University of Singapore, Singapore), Integrative Vessel Analysis (IVAN; University of Wisconsin, Madison), and Vascular Assessment and Measurement Platform for Images of the Retina (VAMPIRE; VAMPIRE group)^1^^–^^3^ can provide a thorough description of the retinal vascular network with geometric parameters (i.e., arteriolar and venular caliber, tortuosity, and fractal dimension [FD]).^4^^–^^7^ These quantitative retinal microvascular biomarkers were shown to be associated with a long-term risk of mortality and ischemic stroke (narrower arterioles and wider venules),^8^^–^^10^ coronary heart mortality (impaired FD),^11^ cardiovascular risk factors,^12^^–^^14^ and an increased cardiovascular risk profile (suboptimal retinal vascular network).^15^ Thus, retinal microvascular biomarkers based on fundus photographs could provide insights into vascular systemic disease. This field of research can be described as oculomics.^16^

These semiautomated software applications require human, trained operators and involve time-consuming interventions for vessel segmentation correction and for artery–vein and optic disc identification, which increases human workload and decreases work reproducibility. To lessen these flaws and to increase the dataset volume, fully automated applications have emerged recently, with new artificial intelligence image-processing software.^17^^–^^21^ Numerous feature-based algorithms have been developed in recent years, such as SIVA DL, Deep Approximation of Retinal Traits, QUantitative Analysis of Retinal vessel Topology and size (QUARTZ), and LUNet.^22^^–^^25^ These deep learning approaches aim to decrease the time required for image analysis and enable the exploration of large fundus datasets. Recently, Zhou et al.^26^ developed an open source deep learning algorithm providing automated analysis of retinal vascular morphology from retinal fundus photographs (automated retinal vascular morphology [AutoMorph]).

Several studies have explored the agreement between semiautomated software and reported inconsistent results.^19^^,^^21^ However, few data are available on the comparison between semiautomated and fully automated software used to extract retinal microvascular biomarker data.^27^ These investigations are mandatory for the generalizability of the results and for fundus-based pooled data analysis. To the best of our knowledge, very few data are available on the agreement between SIVA and AutoMorph measurements of retinal microvascular features.

The current study aimed to compare SIVA software and AutoMorph measurements of retinal microvascular biomarkers in a population-based study of adults aged 75 years and older. The secondary objective was to elucidate whether associations between SIVA retinal microvascular biomarkers and systemic variables in the Montrachet Study were consistent when measured by AutoMorph.

## Methods

### Study Population

The Montrachet (Maculopathy Optic Nerve nuTRition neurovAsCular and HEarT diseases) Study is a French population-based study designed to assess the associations between age-related eye diseases and neurological and heart diseases in older participants.^28^ Briefly, the Three-City Study (3C Study) included 9294 non-[institutionalized individuals aged 65 years and older who were randomly selected from the electoral rolls of three French cities (Bordeaux, Dijon [*n* = 4931], and Montpellier). Baseline examinations (January 1999 to March 2001) consisted of a face-to-face interview (recording sociodemographic and lifestyle characteristics as well as medical history), a physical and cognitive examination, and fasting blood sampling.

The Montrachet Study, described in detail elsewhere,^29^ consisted of a comprehensive eye examination (including fundus photography) in the Department of Ophthalmology of Dijon University Hospital, proposed to participants of the 10-year follow-up of 3C-Dijon study. Overall, 1153 participants agreed to take part and were recruited. All 3C and Montrachet participants gave their written informed consent; the ethics committee of the University Hospital of Kremlin-Bicêtre and the local university hospital ethics committee, respectively, approved the study protocols. The present study adhered to the tenets of the Declaration of Helsinki and was registered as 2009-A00448–49.

To be eligible for the present work, individuals had to be participants in the Montrachet Study who had fundus images with measurements of retinal microvascular biomarkers via SIVA software and the AutoMorph algorithm. We excluded participants who had the following characteristics: epiretinal membrane and an ophthalmological condition interfering with the macular or peripapillary retinal vasculature.

### Retinal Fundus Photographs and SIVA Postprocessing

All patients had retinal fundus photographs taken after pupil dilation with tropicamide 0.5% (Thea, Clermont-Ferrand, France), centered on the optic disc, using a fundus camera (TRC NW6S, Topcon, Tokyo, Japan). For each participant, one eye was retained for analysis based on the following criteria: right eye for participants born in even-numbered years and left eye for participants born in odd-numbered years; for single-eye patients, the functional eye was selected; and if retinal fundus photographs were uninterpretable for one eye, the other one was retained.^15^ Retinal fundus photographs were postprocessed using SIVA. Fundus photographs were anonymously sent to the reading center in Yamagata University, Japan. A single trained grader extracted retinal vessel characteristics using SIVA. A fundus photograph was considered uninterpretable if blurred, if zone C could not be analyzed or if the fundus photograph contained fewer than six arterioles and veins. To study the retinal vascular network, the analysis was based on the six largest arterioles and veins from the optic disc center to three successive zones corresponding to 0.5 optic disc diameter to the margin of the optic disc (zone A), 1 optic disc diameter (zone B), and two optic disc diameter (zone C) (Supplementary Fig. S1).^2^ First, geometric retinal vascular features including FD, vascular caliber, tortuosity, and branching angle were collected and measured by SIVA. An algorithm automatically identified the optic disc and automatically extracted vascular structure and tracing. Then, retinal arterioles (drawn in red) and venules (drawn in blue) were identified and drawn. At the end, algorithms were used to compute quantifiable measurements of retinal vasculature. Human manual intervention consisted of correcting the vessel drawings and removing artifacts.

### Vascular Morphology Feature Measurement With AutoMorph

The same retinal fundus photographs were also analyzed by means of the AutoMorph algorithm, a publicly available and open source algorithm (source code download available at https://github.com/rmaphoh/AutoMorph).^26^ The analysis was based on the same standardized zones as SIVA, corresponding with the annulus from 0.5 to 1.0 optic disc diameter from the optic disc margin (zone B) and to the annulus from 0.5 to 2.0 optic disc diameter (zone C).

### Statistical Analyses and Method Comparison

Retinal parameters that appeared to be analyzed by both applications were compared in this study. The features measured by both applications were as follows: the central retinal venular equivalent (CRVE), the central retinal arteriolar equivalent (CRAE), the arteriolar–venular ratio (AVR), and the vessel complexity measured by FD. The vessel tortuosity was provided by distance measurement tortuosity in AutoMorph and simple tortuosity in SIVA.

As a first step in the process, retinal fundus photographs were classified as gradable or ungradable using AutoMorph, according to a grading quality threshold. This threshold of interpretable images can be set from 0 to 1.

Statistical analyses were conducted using SAS software version 9.4 (SAS institute Inc., Cary, NC). Statistical significance was set at a *P* value of less than 0.05. Continuous variables are expressed as medians (interquartile range) or means ± standard deviation according to their distribution. Categorical variables are expressed as numbers (percentage). The agreement between the two methods was assessed graphically using scatterplots and Bland–Altman plots and quantitatively using type 3 intraclass correlation coefficients (ICCs) with their 95% confidence intervals (CIs).^30^ Strength of agreement was classified using the categories suggested by Hahn et al.^31^ Spearman's correlations assessed the external consistency of the two methods used for analysis of the fundus photographs in terms of the strength of associations with systemic variables (age, sex, treatment for systemic hypertension, body mass index, hypoglycemic treatment, cholesterol-lowering treatment, history of cardiovascular disease and stroke, HeartScore, and major adverse cardiac and cerebrovascular events (MACCE score). History of cardiovascular disease, stroke, and MACCE were variables self-reported by the participants.

This study adhered to the Guidelines for Reporting Reliability and Agreement Studies (GRRAS).^32^

## Results

**Figure 1.**
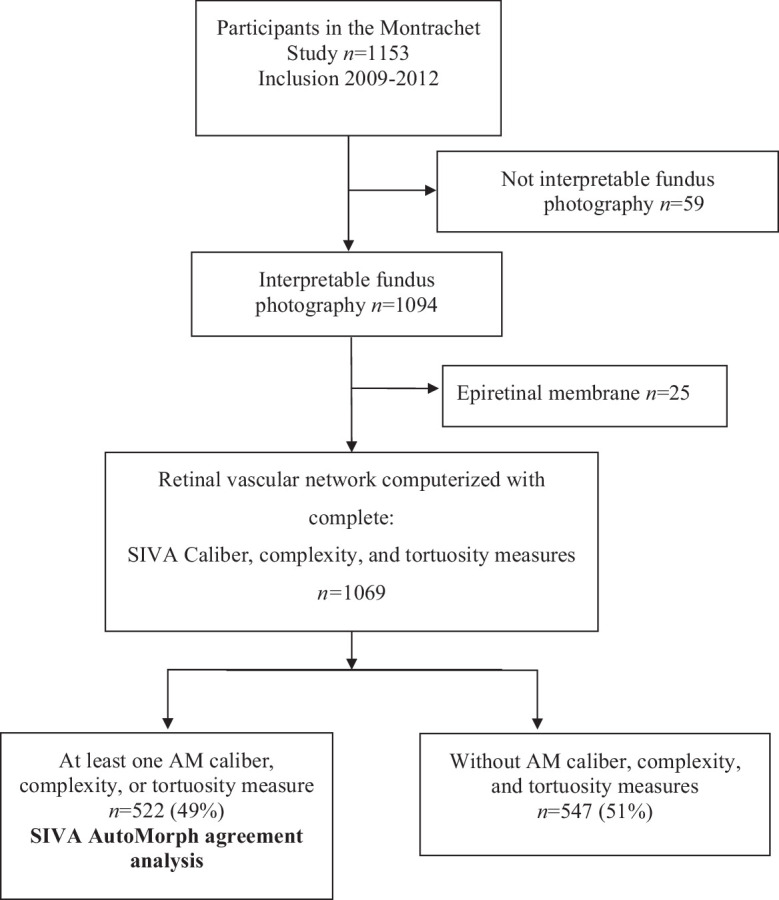
Flow chart of the method comparison study between Montrachet SIVA and AutoMorph.

Overall, 1069 fundus photographs were gradable with SIVA software in the Montrachet Study (Fig. 1). The AutoMorph algorithm analyzed 315 fundus photographs, with a threshold of 0.25. With sequential modification of the threshold, 522 fundus photographs were graded using a threshold of 0.50, 0.75, and 1.00. The lowest rejection rate was 51.17%. The images with uneven illumination and blurry conditions were rejected by AutoMorph. The mean age of the participants analyzed with both SIVA and AutoMorph was 80.04 ± 3.94 years and 69% were female. Table 1 presents the characteristics of the Montrachet Study participants at their inclusion in the study compared with participants with AutoMorph analysis (*n* = 522) and participants without AutoMorph analysis (*n* = 547). There was no statistically significant differences between the two groups, except in sex, mean systolic blood pressure, and rate of stroke.

**Table 1.** Characteristics of Participants With AutoMorph Analysis at Their Inclusion in the Montrachet Study (*n*) Compared With Participants With no AutoMorph Analysis (*n*′)

	Participants With AutoMorph Analysis (*n* = 522)	Participants With No AutoMorph Analysis (*n*′ = 547)	*P* Value
Age, mean ± SD, years	80.04 ± 3.94	80.04 ± 3.75	0.99
Sex			**0.0002**
Male	161 (31)	230 (42)	
Female	359 (69)	316 (58)	
Education			0.54
Primary education	74 (14)	68 (12)	
Lower secondary education	219 (42)	248 (45)	
Upper secondary education	111 (21)	103 (19)	
Tertiary education	116 (22)	125 (23)	
Smoking status (*n* = 482 and *n*′ = 503)			0.073
Non-smokers	290 (56)	276 (50)	
Smokers <10 years	23 (4)	17 (3)	
Smoker ≥10 years	169 (32)	210 (38)	
Treatment for systemic hypertension(*n* = 495 and *n*′ = 519)			0.68
No	164 (31)	182 (33)	
Yes for <10 years	138 (26)	148 (27)	
Yes for ≥10 years	193 (37)	189 (35)	
Systolic blood pressure, mean ± SD, mm Hg	139.02 ± 16.46	141.37 ± 17.15	**0.023**
Body mass index, mean ± SD, kg/m^2^	25.36 ± 3.64	25.58 ± 3.48	0.30
Hypoglycemic treatment(*n* = 474 and *n*′ = 483)			0.39
No	414 (79)	422 (77)	
Yes for <10 years	24 (5)	32 (6)	
Yes for ≥10 years	36 (7)	29 (5)	
Cholesterol-lowering treatment(*n* = 500 and *n*′ = 521)			0.95
No	159 (30)	162 (30)	
Yes for <10 years	90 (17)	97 (18)	
Yes for ≥10 years	251 (48)	262 (48)	
Medical history at Montrachet inclusion			
CVD (*n* = 500 and *n*′ = 518)			0.17
No	471 (90)	472 (86)	
Yes <10 years ago	20 (4)	31 (6)	
Yes ≥10 years ago	9 (2)	15 (3)	
Stroke (ischemic and hemorrhagic) (*n* = 513and *n*′ = 539			**0.032**
No	484 (93)	522 (95)	
Yes <10 years ago	5 (1)	7 (1)	
Yes ≥10 years ago	24 (5)	10 (2)	

CVD, cardiovascular disease; SD, standard deviation.

Data are presented as number (percentage) of participants unless otherwise indicated. Bold values indicate statistically significant results at *P* < 0.05.

**Figure 2.**
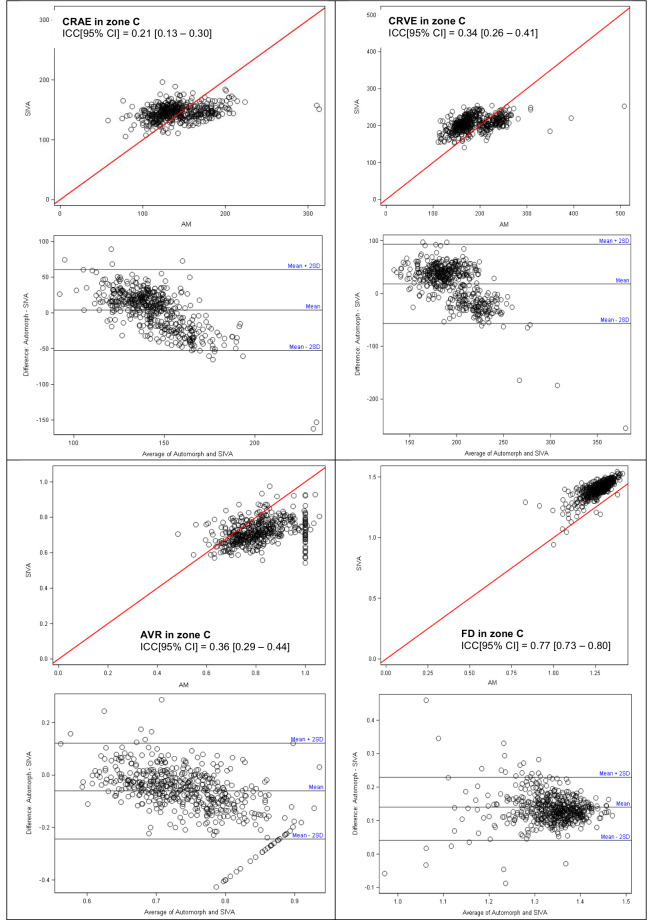
Bland–Altman and scatter plots illustrating correlation of retinal vessel parameter measurements in zone C between SIVA and AutoMorph.

**Figure 3.**
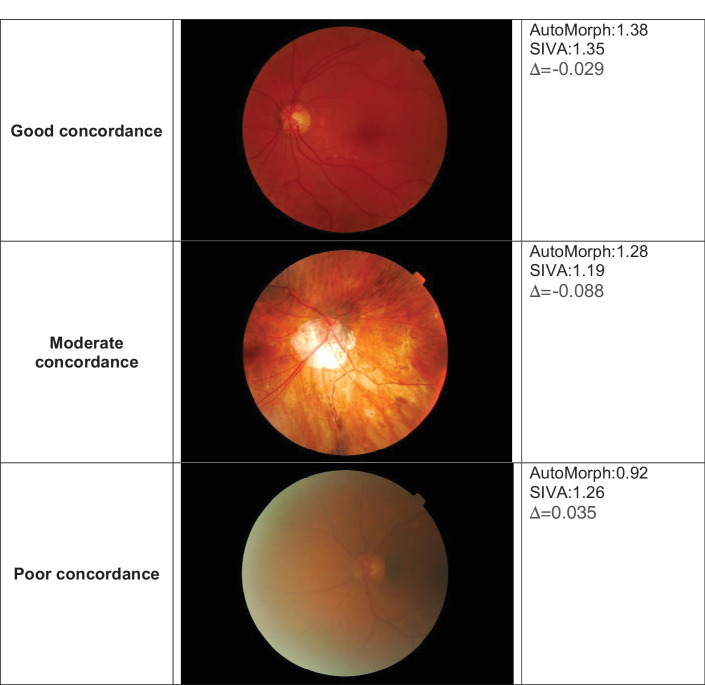
Fundus photography examples illustrating concordance in FD between SIVA and AutoMorph.

The strength of agreement between SIVA and AutoMorph analyses (Table 2) was moderate to good for the FD of arterioles (ICC, 0.47; 95% CI, 0.40–0.53), of veins (ICC, 0.53; 95% CI, 0.46–0.59), and of all vessels (ICC, 0.77; 95% CI, 0.73–0.80). The strength of agreement was poor to slight for all retinal caliber parameters (for CRAE: ICC, 0.23; 95% CI, 0.14–0.32] in zone B and ICC, 0.21; 95% CI, 0.13–0.30] in zone C; for CRVE: ICC, 0.34; 95% CI, 0.26–0.42] in zone B and ICC, 0.34; 95% CI, 0.26–0.41] in zone C). For AVR, there was also a poor to slight agreement in zone B (ICC, 0.32; 95% CI, 0.24–0.40) and in zone C (ICC, 0.36; 95% CI, 0.29–0.44). Figure 2 presents the Bland–Altman and scatter plots illustrating the relationship between AutoMorph and SIVA measurements for these vascular retinal parameters in zone C. Concentration around the first bisector was better for CRVE than for CRAE. For AVR, AutoMorph tended to give higher ratios than SIVA (by a mean estimation of 0.07 units in range of 0.5–1.0 units), but 95% of the differences were within the precision band. For FD, AutoMorph tended to give systematically lower values than SIVA (by a mean estimation of 0.13 units in a global range of 0.83–1.54 units), with a strong proportionality link and 96% of differences within the precision band. However, there was no agreement for vascular tortuosity parameters between the SIVA and AutoMorph analyses, either for arterioles and for veins (ICC, 0.0022 [95% CI, 0–0.09] for arterioles and ICC, 0.0008 [95% CI, 0–0.09] for veins). There was no proportionality link, a mean difference of −2.5 points in a disproportional range, and more than 40% of the differences were out of the precision band (figures not shown). Figure 3 presents examples of fundus photographs with good, moderate, and poor concordance for FD between AutoMorph and SIVA.

**Table 2.** Retinal Vascular Parameter Agreement Between SIVA and AutoMorph

Vascular Parameters	Fundus Photos (*N*)	AutoMorph Mean ± SD (Range, Min–Max)	SIVA Mean ± SD (Range, Min–Max)	ICC [95% CI]	Agreement Strength
CRAE zone B	436	134.45 ± 25.47	140.30 ± 13.87	0.23 [0.14–0.32]	Poor to slight
		(76.81–286.57)	(93.08–195.16)		
CRAE zone C	476	142.11 ± 29.55	145.37 ± 13.19	0.21 [0.13–0.30]	
		(57.86–313.51)	(93.08–196.39)		
CRVE zone B	457	169.23 ± 36.69	196.29 ± 21.08	0.34 [0.26–0.42]	
		(105.23–454.85)	(107.59–265.92)		
CRVE zone C	495	187.30 ± 41.63	205.52 ± 20.23	0.34 [0.26–0.41]	
		(110.79–507.71)	(127.30–283.73)		
AVR zone B	459	0.81 ± 0.10	0.72 ± 0.07	0.32 [0.24–0.40]	
		(0.48–1.06)	(0.54–0.98)		
AVR zone C	499	0.77 ± 0.10	0.71 ± 0.06	0.36 [0.29–0.44]	
		(0.50–1.00)	(0.55–0.96)		
Artery FD zone C	509	1.07 ± 0.11	1.20 ± 0.07	0.47 [0.40–0.53]	Moderate to good
		(0.12–1.25)	(0.73–1.38)		
Vein FD zone C	515	1.08 ± 0.08	1.17 ± 0.06	0.53 [0.46–0.59]	
		(0.56–1.25)	(0.76–1.36)		
All vessels FD zone C	522	1.27 ± 0.07	1.40 ± 0.07	0.77 [0.73–0.80]	
		(0.83–1.41)	(0.94–1.54)		
Artery distance tortuosity zone C	509	3.76 ± 3.22	1.10 ± 0.02	0.0022 [0–0.09]	None
		(0–20.36)	(1.06–1.27)		
Vein distance tortuosity zone C	515	3.53 ± 3.46	1.09 ± 0.02	0.0008 [0–0.09]	
		(1.04–33.66)	(1.05–1.28)		
All vessel distance tortuosity zone C	522	3.57 ± 2.03	1.10 ± 0.02	0.0018 [0–0.09]	
		(1.07–12.35)	(1.06–1.26)		

SD, standard deviation.

Spearman correlation analyses were performed to assess whether the associations between SIVA software retinal features and systemic conditions in the Montrachet Study were consistent when measured with AutoMorph (Table 3). Most associations between retinal vascular parameters found with SIVA were also found with AutoMorph, such as for age and CRAE (*R1* = −0.26; *P* < 0.0001 and *R2* = −0.14; *P* = 0.001), for age and CRVE (*R1* = −0.18; *P* < 0.0001 and *R2* = −0.12; *P* = 0.007), for history of stroke and CRVE (*R1* = −0.11; *P* = 0.013 and *R2* = −0.092; *P* = 0.037), for systolic blood pressure and AVR (*R1* = −0.129; *P* = 0.0003 and *R2* = −0.13; *P* = 0.002), and for history of stroke and AVR (*R1* = 0.089; *P* = 0.045 and *R2* = 0.12; *P* = 0.007). We also found a notably statistically significant association, but only with AutoMorph, for body mass index and CRAE (*R1* = 0.14; *P* = 0.002) and for body mass index and CRVE (*R1* = 0.14; *P* = 0.002). In contrast, we found a statistically significant association, but only with SIVA, for systolic blood pressure and CRAE (*R2* = −0.139; *P* = 0.001), for body mass index and AVR (*R2* = −0.10; *P* = 0.019), for HeartScore and CRVE (*R2* = 0.10; *P* = 0.021), and for HeartScore and AVR (*R2* = −0.14; *P* = 0.001).

**Table 3.** Association Between Retinal Vascular Parameters Measured With AutoMorph and SIVA and Systemic Variables

	AutoMorph			SIVA		
Systemic Variables	*n*	R1	*P* Value	*n*	R2	*P* Value
Retinal arteriolar caliber, CRAE						
Age	474	−0.26	**<0.0001**	520	−0.14	**0.001**
Sex	474	−0.029	0.53	520	0.074	0.09
Treatment for systemic hypertension	452	−0.081	0.08	495	−0.043	0.33
Systolic blood pressure	474	−0.043	0.36	520	−0.139	**0.001**
Body mass index	474	0.14	**0.002**	520	−0.037	0.39
Hypoglycemic treatment	433	0.019	0.70	474	−0.020	0.67
Cholesterol-lowering treatment	457	0.035	0.45	500	0.071	0.12
History of CVD	454	0.017	0.72	500	0.064	0.15
History of stroke	457	−0.061	0.18	513	0.013	0.77
HeartScore	463	0.0019	0.97	509	−0.052	0.24
MACCE	447	−0.044	0.35	493	0.063	0.16
Retinal venular caliber, CRVE						
Age	493	−0.18	**<0.0001**	520	−0.12	**0.0073**
Sex	493	−0.026	0.56	520	0.0017	0.97
Treatment for systemic hypertension	469	−0.041	0.38	495	−0.034	0.45
Systolic blood pressure	493	0.046	0.31	520	0.0074	0.87
Body mass index	493	0.14	**0.0022**	520	0.0074	0.87
Hypoglycemic treatment	450	−0.013	0.78	474	−0.018	0.69
Cholesterol-lowering treatment	474	0.009	0.84	500	0.037	0.41
History of CVD	473	−0.0043	0.93	500	0.028	0.53
History of stroke	486	−0.11	**0.013**	513	−0.092	**0.037**
HeartScore	482	0.083	0.07	509	0.10	**0.021**
MACCE	466	−0.085	0.067	493	−0.034	0.45
Retinal vascular ratio, AVR						
Age	497	−0.076	0.089	520	−0.0084	0.85
Sex	497	0.005	0.91	520	0.057	0.20
Treatment for systemic hypertension	474	−0.096	**0.036**	495	−0.050	0.27
Systolic blood pressure	497	−0.129	**0.0003**	520	−0.13	**0.002**
Body mass index	497	−0.0021	0.96	520	−0.10	**0.019**
Hypoglycemic treatment	453	0.093	**0.048**	474	−0.015	0.74
Cholesterol-lowering treatment	479	0.017	0.71	500	0.007	0.88
History of CVD	477	0.027	0.56	500	0.039	0.38
History of stroke	490	0.089	**0.045**	513	0.12	**0.007**
HeartScore	486	−0.089	0.052	509	−0.14	**0.001**
MACCE	470	0.080	0.082	493	0.11	**0.016**
FD						
Age	520	−0.090	**0.041**	520	−0.13	**0.004**
Sex	520	0.037	0.40	520	0.039	0.37
Treatment for systemic hypertension	495	0.040	0.37	495	0.016	0.72
Systolic blood pressure	520	−0.024	0.58	520	−0.064	0.14
Body mass index	520	−0.029	0.50	520	−0.021	0.63
Hypoglycemic treatment	474	−0.097	**0.035**	474	−0.031	0.50
Cholesterol-lowering treatment	500	−0.016	0.72	500	−0.0009	0.98
History of CVD	500	0.013	0.76	500	−0.042	0.35
History of stroke	513	0.034	0.44	513	0.025	0.57
HeartScore	509	−0.064	0.15	509	−0.040	0.37
MACCE	493	0.034	0.46	493	−0.007	0.86

CVD, cardiovascular disease; MACCE, major adverse cardiovascular events; R1, Spearman correlation coefficient for AutoMorph; R2, Spearman correlation coefficient for SIVA.

Bold values indicate statistically significant results at *P* < 0.05.

## Discussion

In this study, we conducted an in-depth comparison between two different applications to measure retinal microvascular biomarkers from retinal fundus photographs in a dataset of older adults. The SIVA application, a semiautomated software, enabled the analysis of more images than AutoMorph, which rejected 51.17% of the fundus photographs after the image quality grading process. In this specific dataset, AutoMorph seemed to be more sensitive to image quality, excluding a large proportion of retinal fundus photographs analyzed by SIVA. With SIVA, the quality analysis and rate of rejection are determined by manual human intervention, whereas in AutoMorph this step is automated. With AutoMorph, the threshold of interpretable images can be modified from 0 to 1. In this study, various thresholds were tested in a prior analysis to choose the lowest possible threshold for obtaining the highest number of analyzed images. It should be mentioned that there was no improvement in the rejection rate above the 0.50 threshold in this dataset.

In a study based on UK Biobank data analyzed with automated quantification of retinal vessel morphometry using QUARTZ software, 28.4% of the images were labeled as inadequate.^33^ The study confirmed the high rate of uninterpretable images when using automated analysis algorithms. It remains important to know the target population characteristics of the dataset used to train and validate the algorithm, because this factor could explain a high rejection rate in another target population.

Interestingly, in the study based on UK Biobank data analyzed with VAMPIRE, comparing groups with and without analyzable photographs showed that advancing age was associated with a decreased proportion of patients with analyzable photographs, whereas cardiovascular diseases or cardiovascular risk factors did not have a major influence on image quality.^34^ Our results were not in line with these findings, because older age was not statistically significant in our analysis.

AutoMorph and SIVA had a moderate to good agreement regarding vascular complexity evaluated by FD and a poor to slight agreement regarding vascular caliber (CRAE, CRVE, and AVR). However, vascular tortuosity was not well-correlated between AutoMorph and SIVA. We could, therefore, assume that the tortuosity measurement methods were different between the two algorithms. Indeed, in AutoMorph the vascular tortuosity was measured in three different ways—distance tortuosity, squared curvature tortuosity, and tortuosity density—whereas in SIVA only one method was provided with curvature tortuosity.^3^^,^^9^^,^^35^^,^^36^ More harmonized measurement protocols for geometric vascular biomarker could help to reach better agreement.

Moreover, there were discrepancies between the Automorph and Montrachet datasets that could explain such poor agreement. Indeed, the Automorph algorithm was tested and validated in datasets with younger participants.^37^^–^^45^ Fundus photograph device specificity, image compression, image format, or media opacity (lens status) could also explain part of this difference. Table 4 summarizes the population characteristics that could potentially influence retinal microvascular biomarker analysis. Further studies should investigate at which level the heterogenicity of images impact automatic software for a chosen target population.

**Table 4.** Characteristics of Patients in the Montrachet Cohort and Datasets Used for AutoMorph Training and Validation

Dataset	Origin	Device	Age, Years
Montrachet	European	TRC NW6S (Topcon, Tokyo, Japan)	80.04 ± 3.94
AutoMorph			
EyePACS-Q-test^37^	USA	A variety of imaging devices, including DRS (CenterVue, Padova, Italy); iCam (Optovue, Fremont, CA); CR1/DGi/CR2 (Canon, Tokyo, Japan); Topcon NW 8 (Topcon, Tokyo, Japan)	56 ± 10
DRIVE^38^	Netherlands	Canon CR5 non-mydriatic 3CCD camera with FOV equals to 45 degrees	25–90
STARE^39^	USA	TRV-50 fundus camera (Topcon)	Not reported
CHASEDB1^40^	UK	NM-200D handheld fundus camera (Nidek, Aichi, Japan)	9–10
HRF^41^	Germany and Czech Republic	CF-60UVi camera (Canon)	Not reported
IOSTAR^42^	Netherlands and China	EasyScan camera (i-Optics, Rijswijk, Netherlands)	Not reported
LES-AV^43^	Not reported	Visucam Pro NM fundus camera (Carl Zeiss Meditec, Jena, Germany)	71.36 ± 9.98
DRIVE-AV^38^	Netherlands	Canon CR5 non-mydriatic 3CCD camera with FOV equals to 45 degrees	25 to 90
HRF-AV^41^	Germany and Czech Republic	CF-60UVi camera (Canon)	Not reported
REFUGE^44^	China	Visucam 500 fundus camera (Zeiss) and CR-2 camera (Canon)	Not reported
GAMMA^45^	China	Visucam 500 fundus camera (Zeiss) and CR-2 camera (Canon)	Not reported

Based on a similar methodology to our work, a recent study focused on comparing IVAN, a semiautomated algorithm, and the Retina-based Microvascular Health Assessment System (RMHAS), an automated deep learning algorithm.^27^ The ICCs between IVAN and RMHAS were higher than in our study, ranging from 0.42 for AVR to 0.76 for CRVE. This result could be explained by the different age groups included. The mean age in the study comparing IVAN and RHMAS was 48.88 ± 8.38 years, whereas participants in our study were older, with a mean age of 80.04 ± 3.94 years. Thus, we could assume that there were more retinal fundus photographs with interfering retinal anomalies or with more artifacts in the Montrachet dataset, which can lead to a more difficult segmentation and a lower ICC. As presented in Figure 3, concordance between SIVA and AutoMorph seemed to decrease as image quality deteriorated.

Associations of age, history of stroke, and systolic blood pressure with retinal microvascular biomarkers remained consistent with both SIVA and AutoMorph. This point is crucial for future pooled dataset analyses based on fundus photographs. Similar comparisons are needed to confirm that the associations between retinal microvascular biomarkers and systemic vascular features are independent of the fundus image analysis strategy (semiautomated or fully automated). Moreover, the comparisons are also needed to confirm that the transition from semiautomated to fully automated algorithms is coherent with previous studies.

A recent study showed poor stability between intervisit and intravisit retinal parameter measurements by AutoMorph from fundus photographs.^46^ This instability was even more marked for parameters derived from retinal vascular segmentation, such as vascular tortuosity and FD, than for those derived from optic nerve segmentation. In our study, patients were not retested on several fundus photographs to compare the stability of the parameters studied. It is conceivable that image quality may influence these parameters, which was found to have a moderate effect in the study by Giesser et al.^46^ The path to validating retinal parameters as biomarkers of systemic pathologies will require stabilization of these parameters.

The automated feature could make AutoMorph more suitable for analysis of big datasets compared with SIVA. In addition, human involvement in vessel segmentation and identification of the arterioles, veins, and optic disc can lead to potential errors and differences in interpretation if various operators are involved in the data analysis.

This study provided further external validation for the automated analysis of retinal fundus photographs by AutoMorph.

We acknowledge some limitations to this study. First, a small number of fundus images was analyzed compared with other larger studies. Second, SIVA Deep Learning analysis could improve our understanding of limited agreement between semiautomated and automated algorithms. Third, this study was based on a Caucasian population aged more than 75 years, which limits the generalizability of these results.

Further method comparison studies are needed to compare the technical aspects of these imaging software with a larger dataset of sufficient image quality.

## Conclusions

In this study comparing SIVA and AutoMorph, we found a moderate to good correlation for vascular complexity analysis and a poor to slight correlation for vascular caliber analysis, but no agreement on the tortuosity parameters, with a substantial image rejection rate. Although AutoMorph presented promising results in the fully automated analysis of retinal photographs, further research is needed to promote interchangeability and pooled analyses using this software in a large-scale population-based study.

## Supplementary Material

Supplement 1
