# Supplementary material for: Retinal Microvascular Biomarker Assessment With Automated Algorithm and Semiautomated Software in the Montrachet Dataset
Source: Transl Vis Sci Technol. 2025 Mar 12;14(3):13. doi: 10.1167/tvst.14.3.13 (PMC11918093; doi:10.1167/tvst.14.3.13)
Supplement: Supplement 1 [file tvst-14-3-13_s001.docx]

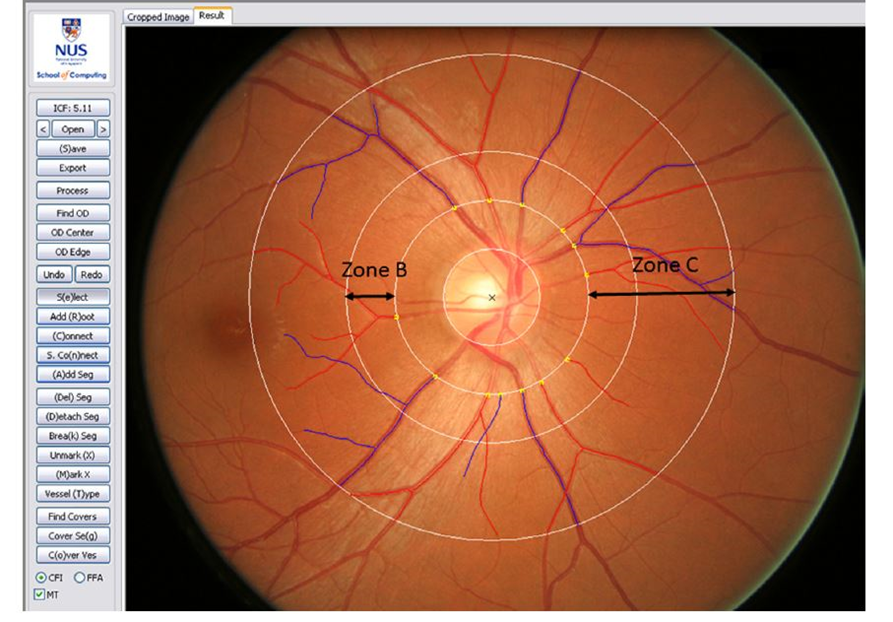


**Zone A**

Supplementary Figure S1. Retinal fundus photograph illustrating Zona A, B and C delimitation and vessels drawings in SIVA.
